# Primary maxillary sinus carcinosarcoma with multidisciplinary management: a case report with 4 years follow-up and literature review

**DOI:** 10.1186/s12903-022-02604-5

**Published:** 2023-02-14

**Authors:** Jiajia Li, Shaohai Wang, Xiufa Tang, Lin Que, Wenzhe Han, Bo Yu

**Affiliations:** 1 Department of Oral and Maxillofacial Surgery, Shanghai East Hospital, Tongji University School of Medicine, 150 Jimo Road, Shanghai, 200120 China; 2 Department of Head and Neck Oncology, State Key Laboratory of Oral Diseases and National Clinical Research Center for Oral Diseases, West China Hospital of Stomatology, Sichuan University, NO.14, 3rd Section of Ren Min Nan Rd., Chengdu, 610041 Sichuan China; 3 Department of Oral and Maxillofacial Surgery, West China Hospital of Stomatology, Sichuan University, Chengdu, Sichuan China

**Keywords:** Maxillary sinus, Carcinosarcoma, Immunohistochemistry, Radiotherapy, Chemotherapy, Prosthetics

## Abstract

**Background:**

Primary maxillary sinus carcinosarcoma (CS) is an extremely rare malignant tumor characterized by biphasic histologic components, lack of standardized treatment, high recurrence rate, and poor prognosis. This paper presents a case of primary maxillary sinus CS and its treatment.

**Case presentation:**

A 39-year-old female patient complained of right facial pain and maxillary teeth numbness on March 21, 2018. Computed tomography examination revealed a malignant mass with osteolytic destruction. Preoperative biopsy suggested sarcomatoid carcinoma or CS. A total right maxillectomy under general anesthesia was performed on April 12, 2018. The final staging was T3N0M0 (ACJJ 2019). Postoperative radiotherapy and chemotherapy were performed. On May 26, 2018, the patient received the first cycle of doxorubicin plus ifosfamide. Two days before radiotherapy, the patient received an intra-oral prosthesis. From June 20, 2018, to August 22, 2018, the patient received concurrent chemoradiotherapy: radiotherapy (60 Gy in 30 fractions) and the second cycle of doxorubicin. Then, the patient received four cycles of doxorubicin plus ifosfamide. The patient was followed for 39 months with no evidence of disease.

**Conclusion:**

Using multidisciplinary therapy, clinical-stage T3N0M0 (ACJJ 2019) maxillary sinus CS may achieve a good prognosis.

## Background

Carcinosarcoma (CS) is a rare biphasic tumor with malignant epithelial and mesenchymal components [1, 2]. CS can occur in different organs, such as the skin, uterus, breast, esophagus, respiratory tract, and parotid glands [3–9], but it is extremely rare in the head and neck [10–13]. Maxillary sinus CS is characterized by rapid progression, strong infiltration, lack of standardized treatment, and poor prognosis [10]. An analysis of paranasal CS has shown a lower 5-year disease-specific survival rate at paranasal sinus than at other sites in the head and neck [14]. The previously reported treatments for CS include surgery, radiotherapy, and chemotherapy [8]. Surgery is the mainstay of CS management because radiotherapy alone and chemotherapy alone result in unfavorable outcomes [8]. A previous case report presented a multimodal treatment in primary hepatic CS (HCS) using surgery and chemoradiotherapy based on doxorubicin and ifosfamide, but the patient did not tolerate the procedure [15]. Studies have shown significant improvement in masticatory function and quality of life (QOL) in patients with total maxillectomy who underwent prosthesis rehabilitation [16, 17].

We report a primary maxillary sinus CS patient who underwent multidisciplinary treatments (including surgery, radiotherapy, chemotherapy, and prosthetics). Primary maxillary sinus carcinosarcoma was defined as a tumor originating in the maxillary sinus and diagnosed as carcinosarcoma. The patient had no history of treatment for maxillary sinus tumors, and metastasis to the maxillary sinus from carcinosarcomas elsewhere in the body was not included. To the best of our knowledge, this is the first patient with maxillary sinus CS with a long survival reported so far.

## Case presentation

**Fig. 1:**
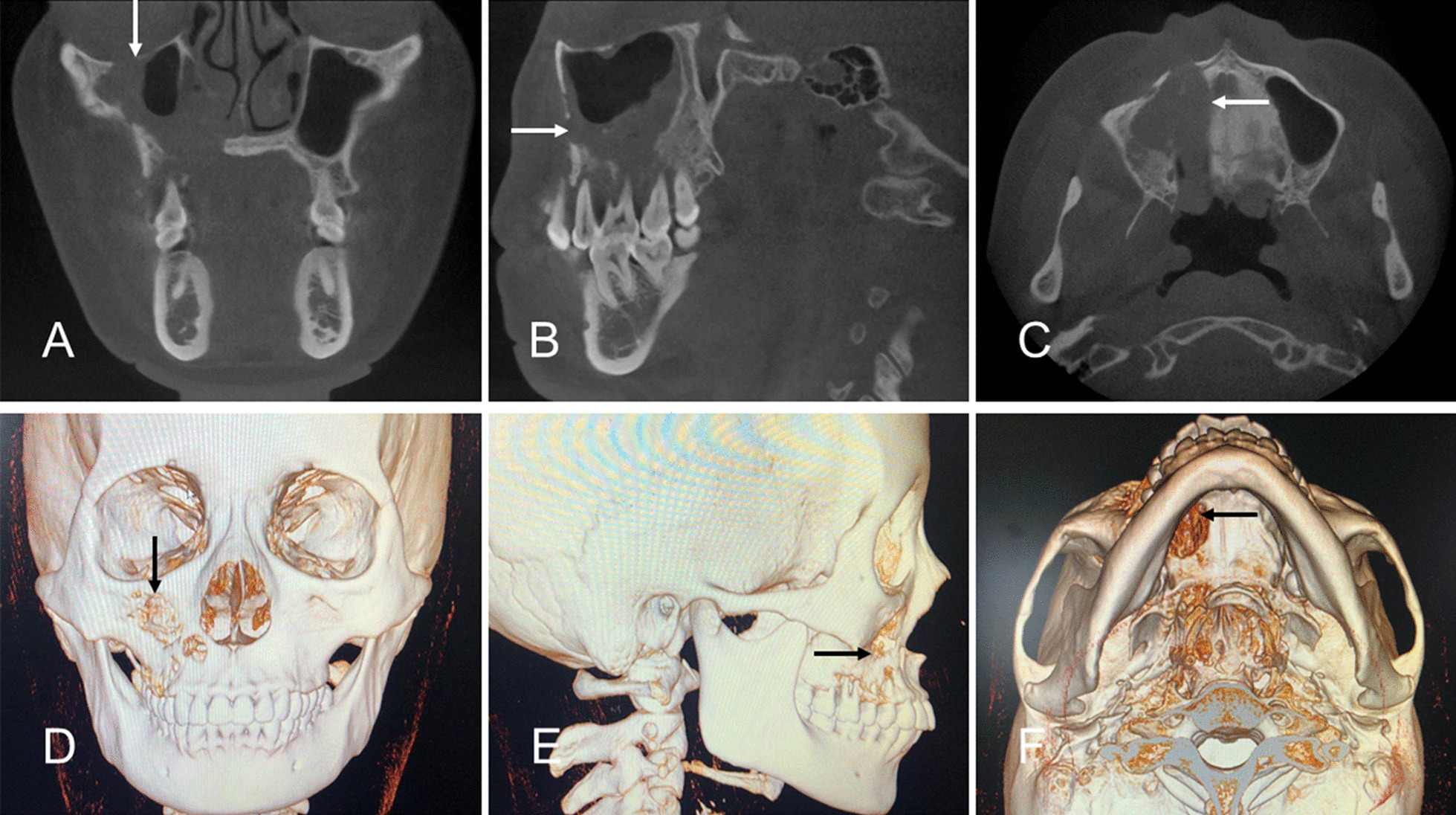
**A**–**C** Cone-beam computed tomography (CBCT) showing the neoplasm (arrows). **D–F** The reconstructed bone destruction (arrows)

On March 21, 2018, a 39-year-old female consulted the West China Hospital of Stomatology (affiliated to Sichuan University), complaining of right facial pain for half a year and numbness for 2 months emerging at her right maxillary anterior teeth. The patient reported no history of smoking and drinking. At physical examination, the patient’s right middle face was slightly swollen without clinically enlarged lymph nodes. On March 27, 2018, an intraoral biopsy was performed in the Outpatient Department. After carefully observing the patient’s imaging results before the biopsy, a 1.0-cm incision was made from the buccal vestibule at the right maxillary first molar in the patient’s oral cavity, directly to the lesion tissue and a small amount of tissue was cut for pathological examination. The pathological examination showed a spindle cell tumor with moderate pleomorphism, mitotic features, and a small amount of new bone tissue. Immunohistochemistry showed PCK (+), CK5/6 (individual +), SATB2 (focal +), CDK4 (individual +), SMA (focal +), HCK (focal +), and CK8/18 (+). The biopsy suggested sarcomatoid carcinoma or CS. A diagnosis of a spindle cell tumor of the right maxillary sinus, with sarcomatoid carcinoma or another tumor, was considered, but the diagnosis had to be confirmed using the surgical specimen. Cone-beam computed tomography (CBCT), and three-dimensional reconstruction of spiral CT images showed a right nasal cavity and maxillary sinus mass (about 6 × 5 × 4 cm) with blurred edges, suggestive of osteolytic destruction (Fig. 1A–F).

**Fig. 2:**
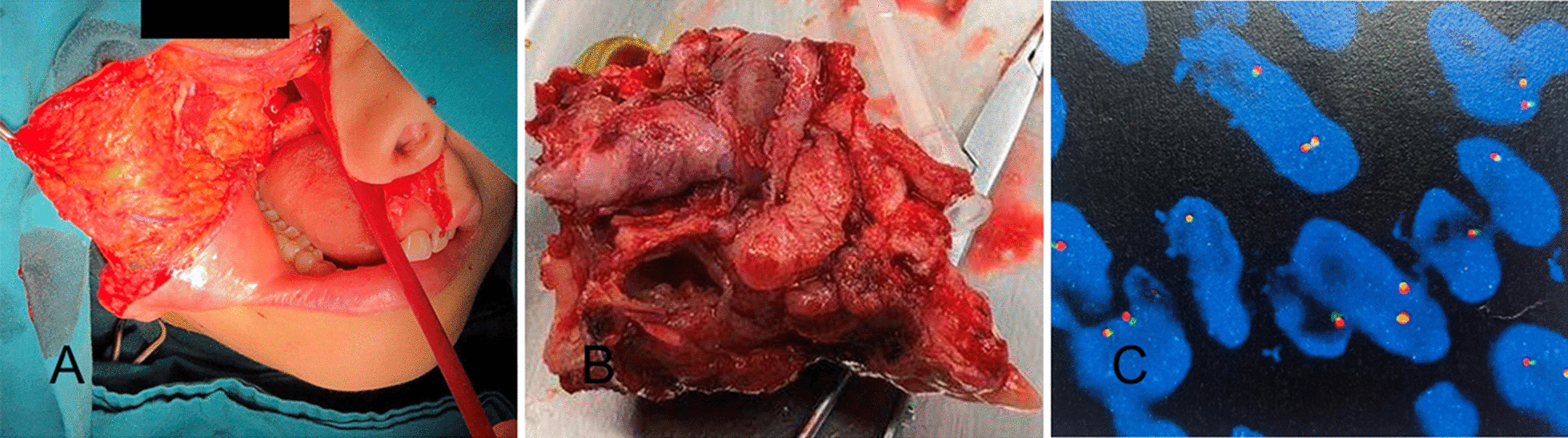
**A**, **B** The total maxillectomy and the mass. **C** The mass was negative for the SS18 (fluorescence in-situ hybridization) gene detection

**Fig. 3:**
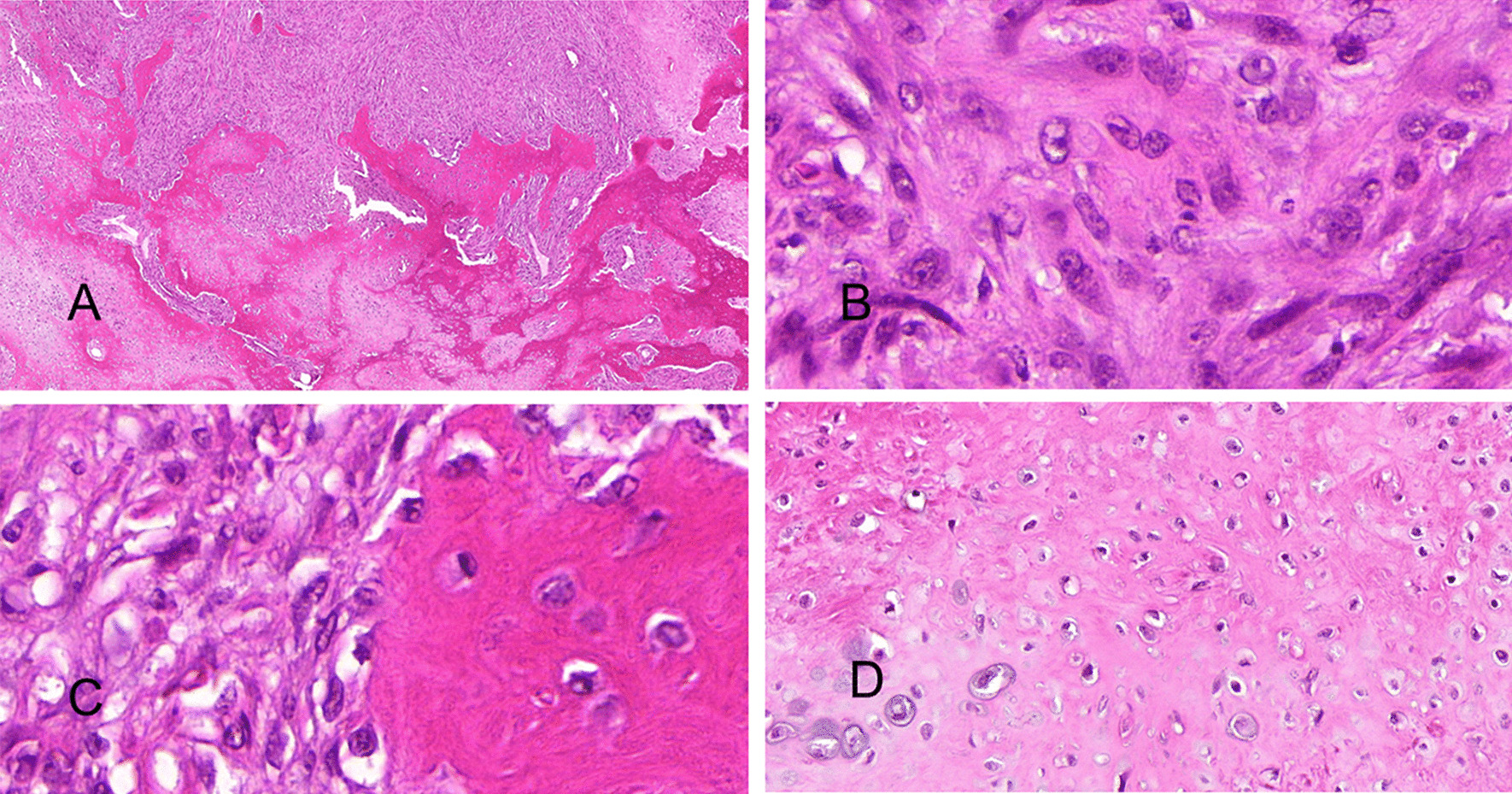
**A** H&E staining showing the epithelial and mesenchymal components. The mesenchymal component contained well-differentiated osteoid and chondroid tissue. **B** The scattered epithelial cells were scattered throughout the mesenchymal tissue. **C** The well-differentiated osteoid tissue. **D** The chondroid tissue and atypical chondrocytes. Original magnification, 40× (**A**), 1000× (**B–D**)

**Fig. 4:**
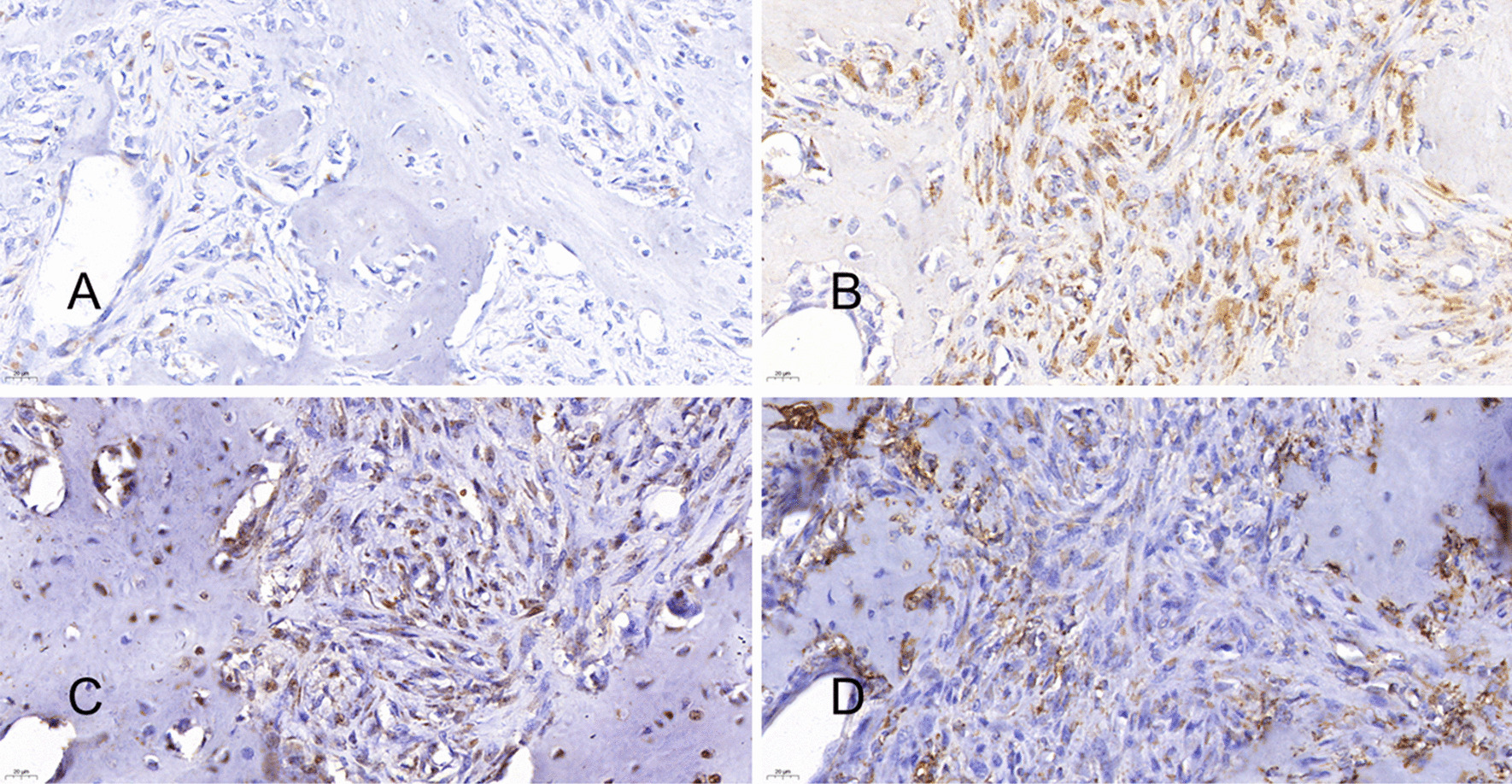
**A**, **B** The epithelial components CK8(+) and CK18(+) by immunohistochemistry. **C**, **D** The mesenchymal component was positive for S100α and vimentin by immunohistochemistry. Original magnification, 400× (**A–D**)

A total right maxillectomy of the right maxilla under general anesthesia was performed on April 12, 2018 (Fig. 2A, B). Postoperative hematoxylin and eosin (H&E) staining showed epithelial and mesenchymal components in the tumor (Fig. 3A–D). Immunohistochemistry (IHC) showed that the epithelial component was positive for cytokeratin 8 (CK8) and CK18, and the mesenchymal component was positive for vimentin and S100α (Fig. 4A–D). SS18 gene, located on chromosome 18q11.2, is found in 95% of synovial sarcomas [18]. Therefore, SS18 gene detection was performed to exclude synovial sarcoma; the results were negative, confirming the diagnosis of CS (Fig. 2C). Based on tumor size and the absence of positive lymph nodes and metastases, the final staging was T3N0M0 (ACJJ 2019).

**Fig. 5:**
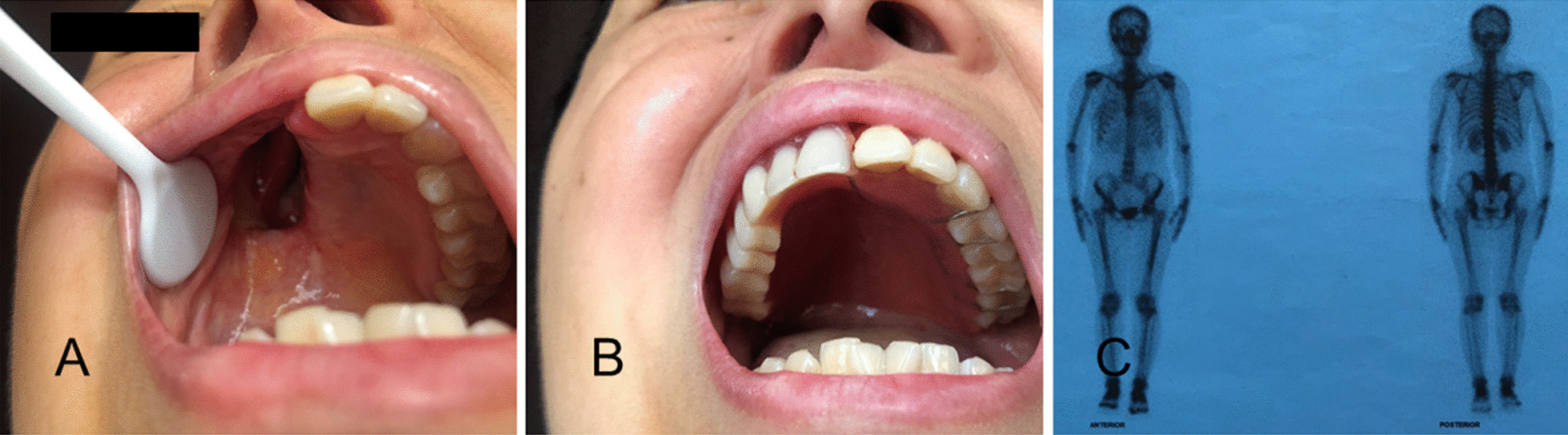
**A–F** Facial and intra-oral images of the patient without and with the intra-oral prosthesis

On May 24, 2018, the patient received an intra-oral prosthesis (Chengdu Koukou Dental Technology Co. Ltd.; Chengdu, Sichuan, China) **(**Fig. 5A–F**)**. On May 26, 2018, the patient received a first cycle of chemotherapy: doxorubicin (100 mg/m^2^; 50 mg/m^2^ per day, day 1–2) plus ifosfamide (12 g/m^2^; 2.5 g/m^2^ per day, day 1–4; 2.0 g/m^2^ per day, day 5). On the third day of the first cycle of chemotherapy, May 28, 2018, the patient developed nausea, stomachache, and general fatigue and refused to continue chemotherapy. The patient did not receive the remaining dose of ifosfamide for cycle 1. The second chemotherapy (doxorubicin 100 mg/m^2^; 100 mg/m^2^ per day, day 1) was started on June 27, 2018. From June 20, 2018, to August 22, 2018, the patient received concurrent chemoradiotherapy: radiotherapy at a total dose of 60 Gy in 30 fractions. The patient received the third cycle of chemotherapy on October 10, 2018 (ifosfamide 2500 mg days 1–4 and 2000 mg day 5; doxorubicin 100 mg day 1, q3w). The fourth cycle was administered on November 22, 2018, the fifth was on December 27, 2018, and the sixth was on January 26, 2019. The four cycles of chemotherapy from the third to the sixth were the same, each lasting 5 days, and the four cycles were separated by 38, 30, and 26 days, respectively. In principle, the treatment plan included a 3-week interval for each treatment cycle, but due to the large side effects of patients on the treatment and the shortage of inpatient beds, the actual interval of each treatment cycle was not equal, and the intervals were longer than the original plan. The patient did not experience grade > 3 toxicity for cycles 2–6. All the subsequent treatment procedures were completed.

We used the modified absorbance method to measure the masticatory efficiency before and after surgery and after the prosthesis was worn for the first time [19]. The average absorbance of masticatory efficiency before and after the prosthesis was worn for the first time was 0.396 abs, and the average absorbance of masticatory efficiency was 0.389 abs after the prosthesis was worn for the first time. The masticatory efficiency increased to 0.506 abs after wearing for one month. The EORTCQLQ-C30 scale [20] was used before and after patients wore the prosthesis and showed that the social function scores were 50 and 83.3, respectively. The self-reported quality of life by the patient was also improved.

Follow-up started on April 23, 2018, when the patient was discharged after surgery. Follow-up was performed at 3 and 6 months after surgery and every 6 months after that. The patient’s general condition was followed up by outpatient visits and phone between visits. Physical examination, maxillofacial magnetic resonance imaging (MRI), and technetium-99 m-methyl diphosphonate (99mTc-MDP) single-photon emission computed tomography/CT (SPECT/CT) showed no recurrence and metastasis for 4 years after surgery. The patient was disease-free on April 20, 2022 (last follow-up). The patient has a good quality of life.

The literature was searched for the reported cases of primary maxillary sinus CS. PubMed, the China National Knowledge Internet, and International Scientific Indexing databases were searched using the keywords “maxillary sinus carcinosarcoma”.

## Discussion and conclusions

The strengths of this case were that it was diagnosed and treated early. The treatments were comprehensive and were based on surgery, chemotherapy, radiotherapy, and prosthetics. The histopathological workup was comprehensive and allowed an accurate diagnosis. In addition, even with a follow-up of > 3 years, the ultimate outcome of this patient is still unknown.

There are many risk factors for maxillary sinus CS occurrence. Some studies showed that maxillary sinus CS might be associated with smoking, alcohol consumption, and radiation [8, 21, 22]. Several somatic gene mutations have been verified in uterine CSs [23–25], including TP53, FBXW7, PIK3CA, PPP2R1A, PTEN, CHD4, KRAS [23], FOXA2 [24], ARID1A, PIK3R1, CTCF, RPL22, INPPL1, and MSH2 [25]. Maxillary sinus CS patients are often admitted to the outpatient clinic because of facial pain and swelling, nasal obstruction, epistaxis, or paresthesia in the affected area [26–30]. Early detection and correct diagnosis are crucial to ensure maxillary sinus CS’s good prognosis [8, 12]. In the case reported here, the patient’s symptoms prompted a biopsy that revealed the malignant nature of the lesion, followed by imaging examinations to characterize the extent of the lesion.

The diagnosis of maxillary sinus CS is mainly based on histopathology and/or immunohistochemistry [10]. Primary CS should be differentiated from spindle cell carcinoma (SpCC) (or sarcomatoid carcinoma), which is composed of carcinoma and malignant spindle cells without mesenchymal components [7, 15, 30, 31]. SpCC is a controversial type of epithelial carcinoma [32]. The characteristics of a true CS are intermixed with biphasic histologic components (pleomorphic epithelial and heterologous mesenchymal components) [15, 24]. The epithelial component includes squamous cell carcinoma, adenosquamous carcinoma, and basal cell carcinoma [11, 27–30]. The mesenchymal component comprises muscle, bone, cartilage, and/or others [14, 30]. In the case presented here, the maxillary sinus CS showed epithelial and well-differentiated mesenchymal components (osteoid and chondroid tissues) (Fig. 3). The diagnosis was demonstrated by postoperative SS18 gene testing, histopathology, and immunohistochemistry. According to the available data, the treatment of SpCC might be different from that of CS [33]. Indeed, a case of inoperable esophageal SpCC showed no response to platinum-based chemoradiotherapy, but neoadjuvant immunotherapy was successful, and the tumor could be removed.

The literature was searched for the reported primary maxillary sinus CSs (Table 1). Consistent with the results of Hasnaoui et al. [34], this review also showed a high percentage of males (57.1%) compared with females (42.9%). The mean age was 59.6 years (range, 39 to 80 years), among which 57.1% were ≥ 60 years, and 42.9% were < 60 years. The combination of surgery, radiotherapy, and chemotherapy represented the largest number of therapeutic regimens (50.0%). The prognosis of the patients was poor, and the local recurrence rate was as high as 71.4%. Of all the cases, only the case reported here survived > 3 years after surgery.

**Table 1:** Primary maxillary sinus CSs in a review of the reported literature

Case no.	Age (years)	Sex	Stage	Treatment	Outcome	Author, year
1	62	F	NR	Surgical resection and RT	LR and metastasis, DWD ~ 1 year after presentation	Meyer and Shklar [38]
2	71	M	T4N0M0	Preoperative RT + TM + removal of eye	Death due to postoperative intracerebral abscess	Feinmesser et al. [26]
3	57	F	NR	Neoplasm excision, ethmoidectomy and turbinectomy	LR 5 months after surgery	Hafiz et al. [21]
4	60	M	T3N0M0	TM + RT + CT	LR, DWD 2 months after operation	Sonobe et al. [28]
5	53	M	T4N0M0	TM + craniofacial resection + RT + CT	LR, free of disease 9 months after repeat resection	Shindo et al. [27]
6	69	F	NR	RT + CT + surgical resection	9 months	Okada et al. [39]
7	80	F	T3N0M0	TM + RT + 2nd operation	LR, death 3 months after the second operation	Sanabre et al. [10]
8	47	M	NR	PM + RT	LR, death 1 year after first operation due to psychologic disorder	Furuta et al. [30]
9	60	M	T3N0M0	TM + RT + CT	LR, FL	Moon et al. [29]
10	61	M	T4aN0M0	TM with modified neck dissection	DWD shortly after surgery due to sternal metastasis	Cheong et al. [12]
11	66	M	T3N0M0	PM + RT + CT	LR, DWD 10 months after initial presentation	Ando et al. [11]
12	55	M	T4aN0M0	RT	Death 4 months after initial examination	Hasnaoui et al. [34]
13	54	F	NR	PM + RT + CT	DWD	de Souza Cruz et al. [13]
14	39	F	T3N0M0	TM + RT + CT	Disease-free 3 years after surgery	Present study 2021

*M* male, *F* female, *NR* not reported, *LR* local recurrence, *TM* total maxillectomy, *PM* partial maxillectomy, *RT* radiation therapy, *CT* chemotherapy, *DWD* death with disease, *FL* follow-up loss

Previous studies suggested that multidisciplinary treatment for patients with CS was the first therapy choice, but they reported different prognoses. Prakalapakorn et al. [31] reviewed different treatments for maxillary sinus CSs. They found that only one of the patients who underwent surgery and radiotherapy developed metastasis, while all patients who underwent surgery and chemotherapy without radiotherapy developed metastases. Studies suggested that the carcinoma component of CS might be more sensitive to chemotherapy and radiotherapy than the sarcoma component of CS [15]. Moreover, recurrence and metastasis of CS often occur in the sarcoma component [15]. The sarcoma component in the recurrence and metastatic foci is similar to those in the primary foci, and adjuvant chemotherapy might help control recurrence and metastasis [27]. Chemoradiotherapy has a certain role in controlling the recurrence and metastasis of CS, but the specific role of adjuvant chemoradiotherapy in maxillary sinus CS is unclear [30, 31, 34]. In the case reported here, the patient was given doxorubicin adjuvant concurrent chemoradiotherapy, and the effect was good. Doxorubicin plus ifosfamide is a common regimen to treat osteosarcoma and soft tissue sarcomas [35, 36]. In this case, the patient was given this regimen because of the amount of sarcoma components in the maxillary sinus CS. To our knowledge, doxorubicin and ifosfamide in the treatment of maxillary sinus CS was not reported before.

Prosthetics are available for defects after maxillectomy, especially for large and complex defects [37]. Consistent with Yusa et al. [16], our follow-up showed that the patient’s masticatory function and the self-reported quality of life with the prosthesis were improved compared with before the prosthesis.

A limitation of this case is that the specimen was not large enough for genetic testing, and no meaningful results were obtained. In addition, oral saliva was tested for MET mutations, but the test failed.

In conclusion, this patient with maxillary sinus CS was successfully treated with multidisciplinary therapy and might achieve long-term disease-free survival, suggesting that multidisciplinary therapy might lead to a good prognosis for patients with maxillary sinus CS. Still, more cases are needed to study the therapeutic effect of multidisciplinary therapy and prosthetics in maxillary sinus CS.

## Data Availability

All data generated or analyzed during this study are included in this published article.

## Abbreviations

99mTc-MDP: Technetium-99 m-methyl diphosphonate
CS: Carcinosarcoma
CT: Computed tomography
H&E: Hematoxylin and eosin
IHC: Immunohistochemistry
MET: Mesenchymal-epithelial transition factor
MRI: Magnetic resonance imaging
QOL: Quality of life
SPECT/CT: Single-photon emission computed tomography/CT
